# Mental Derangements

**Published:** 1889-06

**Authors:** G. W. Sherbino


### "MENTAL DERANGEMENTS."

In states of exaltation we find an excited and mild state cured by Opium, Phos., Acon., Tabac. Raging, shouting, laughing, singing have been removed by Verat. Scolding and inclination to destroy by Hyos. Ready inclination to anger, to strike, or to tear one's clothing by Stram. *Mania in the highest degree*, with attempts at destruction and murder, by Bell.

Foolish imagination, by Anacard. Great talkativeness, confused talking of complicated things, by Cup., Opium, and Verat. When attended with active muscular motions, by Stramon. Talkativeness with delivery of speeches, by Lachesis. Thoughtless actions by Verat. and Hyos.; occasionally, by Bell.

Shameful conversation with sexual excitement by Stram. and Verat.

Religious mania has been cured by Verat. and Aurum.

Seeing ghosts and devils by Opium and Cuprum.

Visions of animals by Bell., Hyos., and Opium.

False impressions about one's self and body by Anac., Stram.

Among the states of depression, we find loss of will and power to decide upon any action have been cured by Coccul. and Helleb.

An apathetic state with dullness, indifference, and brooding, and stupid expression, by Baryta., Helleb., and Opium.

The most numerous observations and cures have been made in the forms of melancholy, from dejection of spirits to the highest degree of anxiety and despair.

Depression of spirits by Conium and Petrol. With fear of death by Platina. Depression of spirits by Stram. and Sepia. Anthropophobia by Anacard.

Melancholic condition by Aurum, Ig., Nat., Sepia, and Rhus tox. When attended with weeping and occurring in connection with pregnancy and confinement by Puls. With desire for solitude, and fear of coming to want by Nux and Calc-c.

Feeling of being unfortunate by Verat. When occasioned by child-bed and misfortune by Bry. Melancholy from care and grief by Caust. Anxious solicitude and fear of starving by Sulph. and Calc-c. Fear of being alone by Stram. Of frightful forms and figures by Puls. Anxious conscientiousness by Lycopod. Anxiety about phantoms of the imagination and constant endeavor to fly from them, Bell.

Restlessness and desire to escape by Stram. and Helleb. and other remedies; despair about shattered health and fear of death by Calc.

Despairing anxiety with fear of approaching misfortune, attended with complaining and weeping, by Cuprum. On account of an unhappy position by Verat.

When the mental derangement assumed the form of fixed ideas we find expectation of approaching death during child-bed cured by Aconite.

The idea of having committed a crime with fear of the officers of justice indicates Cuprum and Zinc. When the physician is mistaken for a police officer, Bell.

The illusion of not being in one's own house indicates Opium. The belief of never being able to be happy in one's own house, Arsenicum. Notions about supposed intentional insult with scruples of conscience, Ignat., Nux, and Puls.

Fear of not being saved, Ignat., Sulph., and Calc-c. Fear of coming to want, Bry-alb., Nux, and Calc-c. Notion that one is composed of two persons, Anacardium.

Arsenicum has cured the inclination to suicide with clear consciousness, from an internal frightful anxiety, although the patient was not tired of life, but wished to be watched and restrained.

In delirium tremens a man in second story of a house was restless and anxious. Wanted some one to stay in the room with him. He was fearful that he would do himself harm by jumping from the window. Thirst, drinking little and often. Cured by Arsenicum.

Aurum has cured persons who thought seriously of taking their own lives ; Nux will cure the melancholy with disgust for life, which drives one to commit suicide.

Aurum is most useful when there is a state of discontent with one's self from supposed bad behavior, or when there is excessive conscientiousness with anxiety, agony of heart, and longing for death.

Hellebore has cured a girl who attempted to drown herself. It is homœopathic when one is tired of life, feels unhappy when he sees others enjoying themselves, and is very envious.

Nux is homœopathic when there is anxiety, as if from a bad conscience or anxiety, with palpitation of the heart driving one to commit suicide, or when one regards his condition as insupportable, so that he would rather die. Pulsatilla, disgust for life, with inclination to drown herself. Anxiety in the region of the heart, with inclination to commit suicide. Veratrum has cured a woman who was about to drown herself on account of her unhappy position.

G. W. Sherbino.

